# Nano-Contact Transfer with Gold Nanoparticles on PEG Hydrogels and Using Wrinkled PDMS-Stamps

**DOI:** 10.3390/polym9060199

**Published:** 2017-05-31

**Authors:** Cigdem Yesildag, Arina Tyushina, Marga Lensen

**Affiliations:** Technische Universität Berlin, Nanopatterned Biomaterials, Sekr. TC 1, Strasse des 17. Juni 124, 10623 Berlin, Germany; cigdem.yesildag@tu-berlin.de (C.Y.); arina1984@web.de (A.T.)

**Keywords:** gold nanoparticles, PEG hydrogels, nano-patterning, wrinkled PDMS, nano-contact transfer

## Abstract

In the present work, a soft lithographic process is used to create nanometer-sized line patterns of gold nanoparticles (Au NPs) on PEG-based hydrogels. Hereby nanometer-sized wrinkles on polydimethylsiloxane (PDMS) are first fabricated, then functionalized with amino-silane and subsequently coated with Au NPs. The Au NPs are electrostatically bound to the surface of the wrinkled PDMS. In the next step, these relatively loosely bound Au NPs are transferred to PEG based hydrogels by simple contacting, which we denote “nano-contact transfer”. Nano-patterned Au NPs lines on PEG hydrogels are thus achieved, which are of interesting potential in nano-photonics, biosensor applications (using SERS) and to control nanoscopic cell adhesion events.

## 1. Introduction

Gold nanoparticles (Au NPs) have many interesting and useful properties such as low biological toxicity, conductivity, easy functionalizability, and size- and shape-dependent localized surface plasmons, which can be tuned towards several applications ranging from inorganic electronic devices to spectroscopy, biosensors and biological applications [1]. Among others due to the afore-mentioned properties, Au NPs are highly requested for localized surface plasmon resonance (LSPR) spectroscopy and surface enhanced Raman scattering (SERS) [2,3]. For both of these methods the plasmon couplings among the Au NPs are playing the key role and should be therefore studied more precisely. Correspondingly, several groups are putting effort on nano-scaled patterning or self-organization of Au NPs on different surfaces, for instance for application in nanophotonics [4]. From a biological point of view, studies of cellular adhesion on specifically functionalized Au NPs with nano-scaled patterns have been investigated in recent years. For example, Spatz et al. created Au NPs via the block copolymer micelle nanolithography method, functionalized the Au NPs with surface adhesive peptides (e.g., Fibronectin) and described that the nanometric dimensions of the periodic Au NPs patterns effect the formation of focal adhesions and the composition of the extracellular matrices [5,6,7]. They also investigated cellular adhesion of MC3T3-osteoblasts on c(RGDfK)-thiol-coated Au NPs in the range of 52 and 73 nm, where they proposed that the nanoscopic length scale for integrin clustering and activation highly influences the adhesion behavior [8]. Our own, very recent studies have shown that the cell adhesion of mouse fibroblasts L929 cells even on bare (non-bio-functionalized) Au NPs-coated poly(ethylene glycol) (PEG) based hydrogels was highly increased in comparison to other surfaces like tissue culture polystyrene (TCPS) or glass [9]. That’s why controlling of Au NPs patterns on inert polymeric backgrounds (e.g., PEG) at the nanometer scale, are an indispensable tool for studying and understanding fundamental cellular adhesion processes.

Hydrogels are three-dimensional networks of hydrophilic polymers, which absorb large amounts of water without losing the network structure and without being dissolved in the aqueous solution, similar to organic tissues [10]. In order to understand and control the nano- or micrometer sized phenomena of cell adhesion, migration and tissue organization in nature, nano- or micro-patterns have been fabricated on the surface of the hydrogel materials. Surface patterning of hard substrates like glass, silica or mica are quite easily fabricated by different lithographic or soft lithographic procedures. In order to mimic soft tissue however, it is important to translate the nano- and micro-structures to soft, hydrated biomaterials. That is why we have developed strategies to convey nano-patterns from hard substrates to soft gels.

Especially PEG-based hydrogels are suitable due to their non-toxicity, non-immunogenicity and non-fouling characteristics that offer excellent background properties to study fundamental cellular processes, such as adhesion, migration and proliferation [11]. Hydrogels can be patterned topographically, elastically or chemically, as we and others have reported during recent years. Topographical patterns can be realized via for example replica molding as developed by Whitesides et al. [12] or by simple micro- and nano-molding [13]. With our innovative Fill-Molding in Capillaries (FIMIC) [14] process, elastically patterned PEG hydrogels can be achieved. Other widely used patterning methods based on soft lithography are micro-contact printing (µCP) [15,16] or Micro-Molding in Capillaries (MIMIC) [17,18].

Notwithstanding their great use and potential, these microfabrication techniques inherently produce micrometer-sized features. As mentioned before, in many cases, control of patterns at the nanometer scale is necessary as well. This can be achieved by nanofabrication methods, e.g., electron beam lithography (EBL) [19], dip-pen nanolithography (DPN) [20] or nano-transfer printing (nTP) [21,22] methods. Zheng et al. described a procedure where they could pattern gold colloids on silicon wafers by combining AFM-based nano-oxidation and chemical assembly of colloidal nanoparticles on different surface functions [23]. Wang et al. tuned AFM tips for the direct writing of Au NPs on surfaces using the dip-pen nanolithography technique [24]. Beside the highly precise nanoscale resolution of these types of scanning probe-based lithographic techniques, inherent disadvantages are the high time exposure and the limited working surface area, since conventional AFM instruments usually have maximally 100 μm × 100 μm working lengths. The same concerns can be also inferred for electron beam lithography, which works with high precision and high resolution but with high time exposures and also high energy costs.

As mentioned above, Spatz et al. presented different possibilities for patterning Au NPs on different (hard) substrates at nano- and micrometer scales using the block copolymer micelle nanolithography. Further, for patterning of hydrogels, Ding et al. developed a process where the patterned Au NPs on glass could be transferred onto the surface of hydrogels [25]. Hereby the Au NPs are functionalized with specific linker molecules and are bound to hydrogels surface after contacting with the glass surface. Such a transfer can also be achieved by using specifically functionalized hydrogels [9]. In our recent work, we have been able to transfer Au NPs from silica wafers or glass substrates to hydrogel surfaces with the wet micro-contact deprinting procedure without utilizing any specific linker molecules. The transfer occurs after the hydrogel is swollen in water during the contact with the Au NPs. This process is quite general, yet very versatile and effective, so that we could achieve well-arranged micro-sized line and rectangular patterns of Au NPs on PEG hydrogels. These nano- and micro-patterned hydrogels have been investigated in cell culture and have been found to be very useful for controlling specific cell behavior (see our other contribution to this special issue; [26]).

Not only for cell culture but also for spectroscopic devices; biosensors or nanophotonic materials, precise control over Au NP patterns at the nanometer scale is necessary. To that end, Fery et al. [27,28] used specifically nanometer sized wrinkled polydimethylsiloxane (PDMS) for nano-patterning of Au NPs for controlling of the SERS performance of Au NPs nanostructures on silicon wafers and Madsen et al. used nano-patterned Au NPs arrays to improve light absorption for the enhancement of the efficiency of organic solar cells [29].

In this work, we present a process to create controlled nanometer-sized patterns of Au NPs on PEG based hydrogels. The nano-patterns of Au NPs were achieved by the controlled targeting of the surface-wrinkle sizes of PDMS by tuning the plasma treatment time of the stretched PDMS according to the principles introduced by Whitesides et al. [30] Subsequently, after having obtained the desired wrinkle sizes, the surface of the PDMS was amino-silanized and coated with citrate-capped Au NPs. The Au NPs were bound to the surface of the wrinkled PDMS and were transferred to PEG hydrogels by the “Nano-Contact Transfer” process, in a well-arranged manner, exhibiting nanometer-sized lines.

## 2. Materials and Methods

### 2.1. Chemicals

Tetrachloroaurate trihydrate (HAuCl_4_·3H_2_O), trisodium citrate (Na_3_C_6_H_5_O_7_), poly(ethylene glycol) diacrylate (PEGDA, Mw 575) and 2-hydroxy-4′-(2-hydroxyethoxy)-2-methylpropiophenone (photoinitiator-PI Irgacure 2959) were purchased from Sigma-Aldrich Chemie GmbH (Steinheim, Germany). (3-Aminopropyl)trimethoxysilane (APTMS) was bought from ABCR GmbH & Co. KG (Karlsruhe, Germany). Poly(dimethylsiloxane) (PDMS) and the curing agent are from Dow Corning GmbH (Wiesbaden, Germany). All the chemicals were used without further purification.

### 2.2. Synthesis of Au NPs with Different Sizes

#### 2.2.1. Synthesis of Au NP Seeds

The citrate capped Au NP seeds were synthesized as described by Bastús et al. [31]. Briefly, in a three-necked round bottom flask 2.2 mM of trisodium citrate were dissolved in 150 mL of deionized water and heated for 15 min under vigorous stirring. A condenser was used to prevent the evaporation of the solvent. After boiling had commenced, 1 mL of a solution containing 25 mM of H[AuCl_4_]·3H_2_O was added to the trisodium citrate solution. The resulting pink mixture was kept stirring under reflux for additional 10 min.

#### 2.2.2. Seeded Growth of Au NPs

Immediately after the synthesis of the Au NPs seeds was finished, the reaction was cooled down until the temperature of the solution reached 90 °C. Afterwards, 1 mL of the 25 mM H[AuCl_4_]·3H_2_O solution was injected and the reaction was stirred for 30 min. This process was repeated twice. After the third addition of the precursor, the Au NPs solution was diluted by extracting 55 mL of the Au NP solution and adding 53 mL of deionized water and 2 mL of a solution of 60 mM trisodium citrate. This solution was then used as the seed for the subsequent growing step, repeating the whole process again. The reaction temperature was maintained at 90 °C during the growing steps. In that way depending on the number of growing steps spherical Au NPs with diameters from 20 up to 200 nm are possible to achieve. For detailed information see original paper of Bastús et al. [31]. In this article we worked with 20 nm Au NPs.

TEM characterization can be found in the Supplementary Materials (Figure S1).

### 2.3. Preparation of PDMS-Stamps

#### 2.3.1. PDMS Wrinkling

Wrinkled PDMS structures were done respectively to the procedure of Whitesides et al. [30]. Firstly a PDMS elastomeric mold was prepared. The PDMS mold was prepared by using a mixture of Sylgard 184 silicone elastomer and curing agent (10:1; *v*/*v*). In order to avoid bubbles the mixture was degassed in a desiccator, then casted on flat glass slide and cured 2 h at 120 °C. After the PDMS was cooled down to room temperature, small pieces of PDMS were cut and placed in a stretch apparatus and stretched from one side to 125% of the original sizes of the PDMS pieces. The PDMS were treated with an oxygen plasma (0.2 mbar, 80 keV) for a predefined period of time in the stretched condition. Subsequently after plasma treatment the PDMS pieces were relaxed. During the relaxation process well-arranged nanometer sized wrinkles on the PDMS surfaces were created.

#### 2.3.2. Amino-Silanization of Wrinkled PDMS-Stamps

Subsequently after the PDMS wrinkles were created via plasma treatment, the PDMS-stamps were placed in a desiccator containing two drops of amino-silane agent. Then vacuum was kept and left for 2 h.

### 2.4. Preparation of the PEG Hydrogels

**Figure 1:**
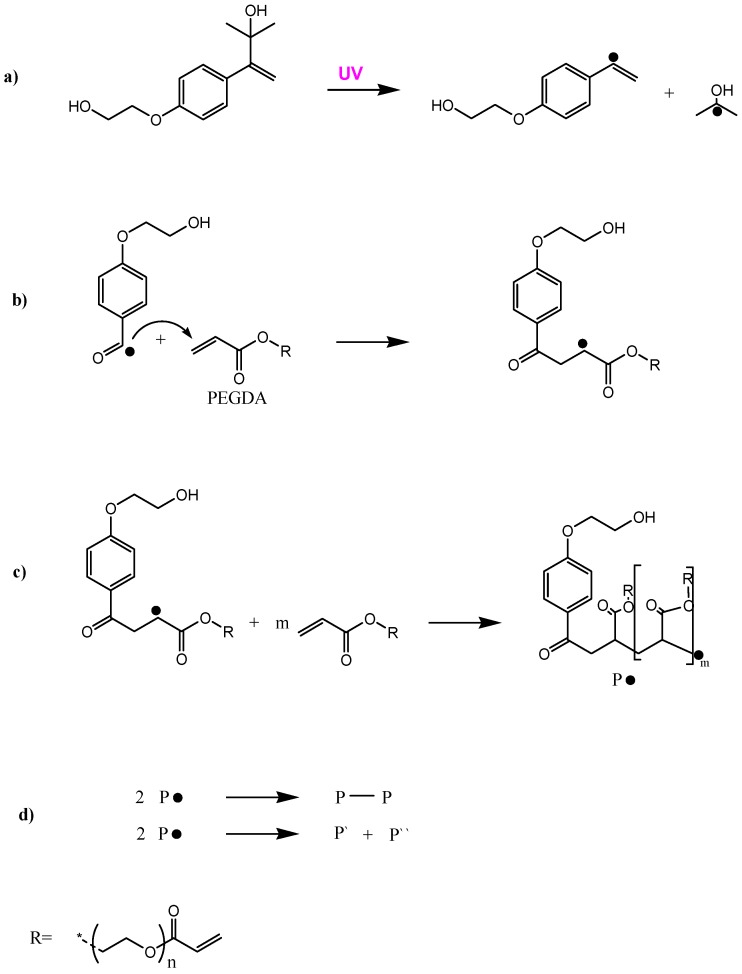
Reaction steps for the photo-polymerization of PEG with terminal acrylate groups with the photoinitiator Irgacure 2959: (**a**) Initiation; (**b**) Propagation; (**c**) Chain-transfer; (**d**) Termination. Image modified from ref. [32].

PEG hydrogels were prepared using the UV-curing process (see Figure 1). For that linear PEG–diacrylate (*M*w = 575 g/mol) was used as precursor and was mixed with 1% of photoinitiator Irgacure 2959. For having a good distribution of the photo-initiator the mixture was sonicated for around 5 min. Then the mixture was dispensed on a glass slide and covered with a thin glass cover slip to achieve a flat hydrogel sample. This liquid mixture was put under UV-light source for 8 min and the glass cover slip was peeled off. The result was a thin, flat hydrogel sample, as a stand-alone film.

### 2.5. Coating of the Wrinkled PDMS-Stamps with Au NPs and Transfer to PEG Hydrogels

**Figure 2:**
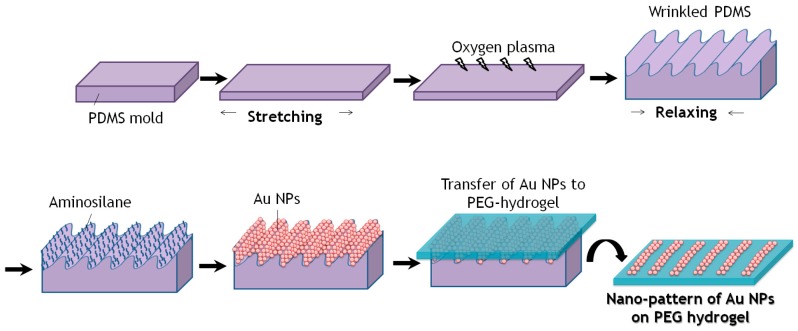
Schematic view of Nano-Contact Transfer of Au NPs from a wrinkled PDMS-stamp onto a stand-alone hydrogel film.

The wrinkled and amino-silanized PDMS was coated with the respectively synthesized Au NPs, which had a concentration of 3.0 nM according to the extinction coefficient of ε = (8.78 ± 0.06) × 108 M^−1^·cm^−1^ [33]. Hereby a droplet of Au NPs solution was dispensed on the wrinkled PDMS-stamp and left for 1 h at R.T. The coated PDMS was then washed rigorously in water and dried with a flow of nitrogen before the subsequent transfer process. After drying, the Au NPs coated PDMS-stamp was contacted firmly with pre-molded PEG hydrogel (see Figure 2).

## 3. Results and Discussion

**Figure 3:**
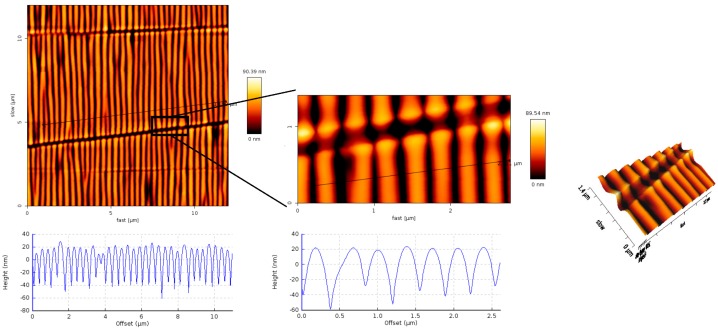
AFM height images and cross-section profiles of the (non-silanized) wrinkled PDMS-stamp and of the horizontal line breaks, or cracks.

First of all, wrinkled PDMS-stamps were prepared via plasma oxidation of smooth PDMS molds in a stretched configuration, and subsequent relaxation of the stamp to the original macroscopic dimensions [30]. By this wrinkling process, periodic and regular nano-line topographies were achieved on the surface of the PDMS-stamps. The characteristic lateral and vertical dimensions appeared to depend in a predictable way on the plasma treatment time and energy, as is demonstrated by atomic force microscopy (AFM) analysis (Figure 3).

**Figure 4:**
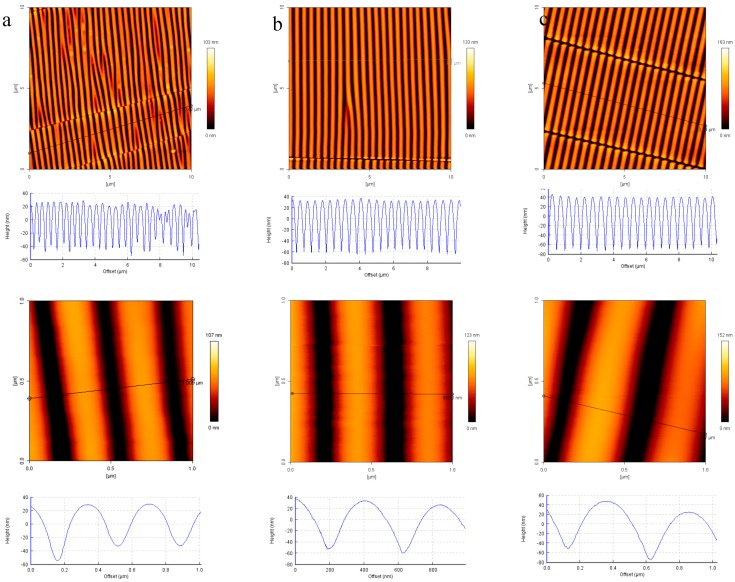
AFM height images and cross-section profiles for different plasma treatment times for PDMS-stamps coated with NH_2_-silane: (**a**) 2 min; (**b**) 5 min; (**c**) 15 min.

In the next step, the surfaces of the wrinkled PDMS-stamps were silanized with amino functionalities (amino-silane; APTMS). For comparison, AFM measurements of the non-silanized PDMS-stamp were performed for the 2 min plasma-treated stamp and no significant different was observed on height images before and after amino-silanization (Figure 4). This similarity is attributed to the high quality of the dense, thin monolayer of the silanes (<1 nm) on the stamp.

The sizes of the wrinkles or nano-topographies were controlled by the plasma oxidation time; in general it can be said that the longer the plasma oxidation time the wider the distances of the line spaces and the deeper the grooves were. The distances of the nano-lines (from hill to hill) varied from around 300 up to around 600 nm by choosing a plasma oxidation time of 2 up to 15 min, which can be seen in the AFM images in Figure 4; for a plasma treatment time of 2 min ~362 ± 61 nm, 5 min ~453 ± 36 nm and for 15 min around ~580 ± 53 nm distances were measured by AFM analysis. For the grooves approximately 60, 80 and 100 nm depths are measured for the respective plasma times from 2 min to 15 min. These details are also plotted in the Supplementary Materials, Figure S2. Besides the vertical nano-lines there were also irregular, perpendicular cracks observable in the AFM images. The AFM height image in Figure 3 shows that the nano-lines close to the cracks were elevated. This was also observable in the other images of the stamps with a bright color appearance of the nano-lines in close contact to the cracks. The millimeter-long cracks are supposed to happen during the relaxation after the regular, nanometric wrinkles have been formed as presented in several literature reports before [30,34,35,36,37].

**Figure 5:**
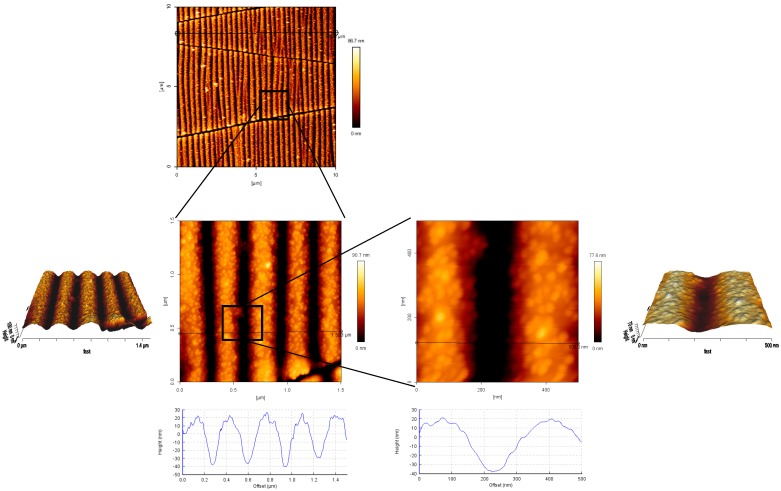
AFM-height images and cross section profiles of 2 min plasma-treated PDMS-stamp after Au NPs coating.

The amino-silanized surfaces were subsequently coated with Au NPs (see Figure 5). For that purpose, the whole surface of the wrinkled PDMS-stamp was covered by a monolayer of Au NPs, while the original wrinkled structure specifications were unaffected (see Figure 5). Coating of the PDMS-stamp with amino-silane layer was crucial for having a good coverage of Au NPs interacting with the surface via electrostatic interactions, and which could be easily transferred to the desired end surfaces. Without amino-silane layer no Au NPs could be seen on the PDMS-stamp.

**Figure 6:**
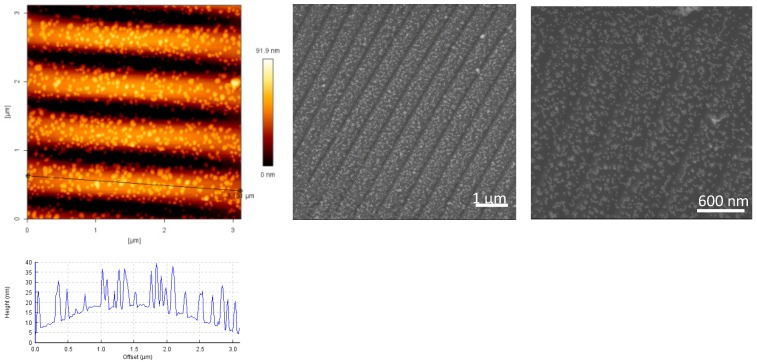
AFM and SEM images of Au NPs distribution on the PDMS‑stamp.

The distribution of the Au NPs on the surface of the PDMS-stamp is shown in the AFM and SEM images in Figure 6. The Au NPs were distributed over the whole PDMS surface with interparticle distances of around 5 up to 100 nm, and a particle density of 7 ± 1 particles/100 nm^2^. The distances of the particles can be further tuned with dilution processes of the Au NPs solutions before coating on the stamp.

After having an Au NPs-coated PDMS-stamp, the Au NPs can be transferred via stamping on different hard surfaces like on Si-wafers or glass, or on softer surfaces like on hydrogels. For the nano-contact printing process, the surface properties of both the stamp and the substrate are crucial in determining the transfer efficiency. The molecules or the particles, which will be transferred, should interact with the stamp with an intermediate affinity; the interaction should be strong enough so that the particles can distribute over and cover the stamp properly but also loose enough to be released after contacting with the surface of the eventual goal substrates. 

In the present work, the interaction of Au NPs with the PEG hydrogels surfaces was likewise important. Here, the Au NPs were stabilized with citrate molecules that provide the Au NPs with a negative surface charge. The amino-groups on the surface of the PDMS-stamp manifest positive charges in aqueous medium, so there were attractive electrostatic interactions.

**Figure 7:**
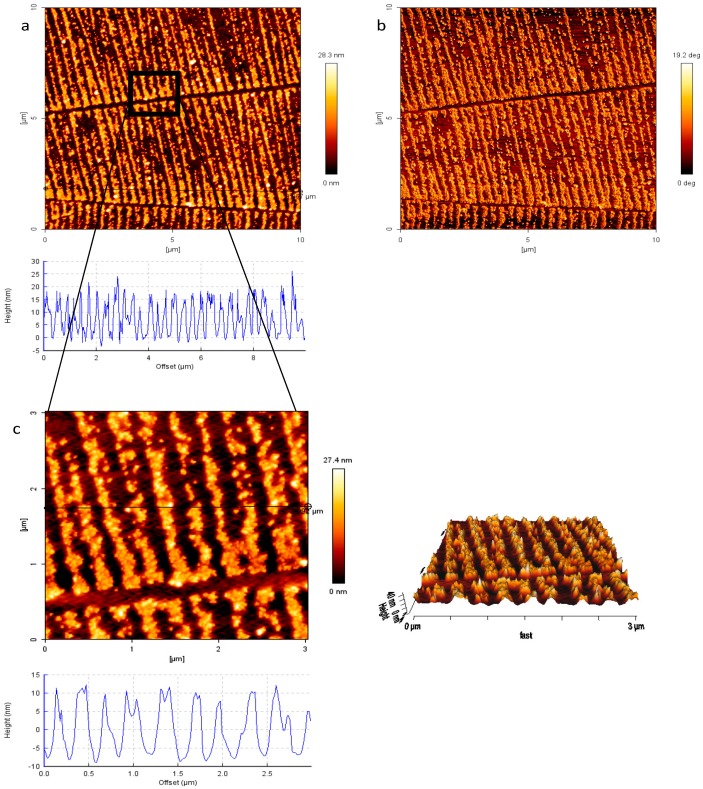
Nano-lines of Au NPs on the surface of PEG hydrogel; (**a**) AFM height image and cross section profile; (**b**) phase image and (**c**) AFM height image and cross section profile of an enlarged view of (**a**).

Au NPs were then transferred onto non-reactive, non-charged hydrogels (Figure 7). It can be observed that the transfer is efficient and sufficient (even though the calculated transfer efficiency is only 50%, vide infra), and the nano-pattern resolution with all the characteristic nano-structures is very accurate. The distances among the lines vary between 300 and 350 nm (Figure 7c) in analogy to the used stamp, which was plasma treated for 2 min (Figure 3). The original nano-pattern and even the features of the cracks were accurately transferred. The height cross-section profiles show a height value of 20 nm, which corresponds perfectly to the size of the used Au NPs. The successful creation of the nanocomposite material (Au NPs–PEG–gel) is further supported by the phase image in Figure 7b, which show two different colors and this contributes to two different materials; these are the soft PEG hydrogel constitutes the flat background and the hard Au NPs, which are sticking out of the surface. In Figure 7 the nano-lines of Au NPs are in some areas loosely packed whereas in other areas they seem quite densely packed. One possible reason for this could be the irregular not completely flat-shape of the stamp close to the cracks, as discussed above. It can be seen that close to the cracks, the Au NPs appear more closely packed and in the more peripheral parts of the nano-lines the density is somewhat decreased (Figure 7a).

**Figure 8:**
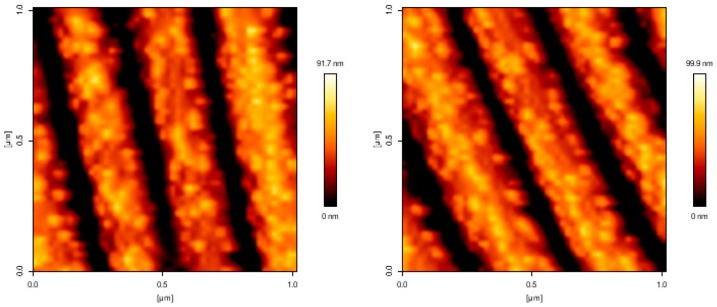
AFM images of Au NPs coated PDMS-stamp after contact to the surface of PEG hydrogel.

As far as the transfer efficiency is concerned, taking a closer look at the Au NPs-coated PDMS-stamp after contact with the PEG hydrogel (see Figure 8) it could be observed that not all of the Au NPs were transferred on the surface of the hydrogel. Obviously, those Au NPs that were mainly located on the top part of the protrusions are more readily transferred to the gel in conformal contact. Roughly, around 50% of the Au NPs are still remaining on the surface of the PDMS-stamp. See Supplementary Materials, Figure S3 for further details.

In order to improve the transfer efficiency, softer PEG hydrogels could be used, which will have a better conformal contact with the stamp surface and peel off the Au NPs more efficiently. Besides the simple physico-chemical properties, also chemical interactions of the hydrogel can be further tuned. The substrate is needed to be more attractive towards the Au NPs than this electrostatic interaction in order to take up the Au NPs from the stamp efficiently. In that case mercapto-functionalized hydrogel substrates will be an option, due to the strong covalent binding affinity of the Au NPs with thiol-functions. Currently, we are exploiting reactive, thiolated hydrogels that have great affinity to Au NPs (see also our other contribution in this special issue; [38]).

Another possibility to increase the transfer efficiency is to cover the surface with the liquid PEG–prepolymer and do the UV-crosslinking directly on the surface of the Au NPs-patterned stamp and peel off the cured hydrogel from the surface by swelling according to our other contribution in this special issue [26]. This would increase the Au NPs transfer efficiency to above 99%. At the same time, the nano-topography would be also copied on the surface of the hydrogel and Au NPs distribution on PEG–hydrogel with nano-topographical structures would be created. For different types of applications, where Au NPs on nano-topographies are required, this would be an interesting option.

Such Au NPs-patterned PEG–hydrogels are also of great interest to control cell adhesion with nanoscopic precision, as we have recently reported [9,26,38]. In the Supplementary Materials, some cell culture results on these novel biomaterials are shown and discussed (Figure S4).

## 4. Conclusions

In this work we presented an easy and fast soft-lithographic method to achieve nano-sized patterns of Au NPs with different sizes on PEG hydrogels without utilizing any specifically prefabricated silicon masters, but rather using only soft, polymeric materials as molds, stamps and substrates.

The pattern sizes were originally controlled via the wrinkled PDMS surface structure, while tuning of the plasma treatment times. In general, it could be stated that the longer the plasma treatment time, the larger are the distances between the grooves of the wrinkles and the deeper are the grooves on PDMS-stamps.

In the second step, the wrinkled PDMS was surface-functionalized with amino-silane molecules and coated with citrate-capped Au NPs. Thus, the Au NPs were bound on the surface of the PDMS via effective, yet non-covalent, electrostatic interactions.

In parallel, smooth and flat films of PEG-based hydrogels were prepared to serve as the versatile and inert, biomaterial goal substrate.

The nano-patterns of Au NPs were subsequently transferred onto the surface of the PEG–hydrogels through contacting; a process that we denote “Nano-Contact Transfer”.

Especially the non-toxicity and non-fouling characteristics of PEG based materials and the size-and shape-dependent optical properties and the low toxicity and make these materials highly useful for biological or biomedical devices, tissue engineering, drug delivery and cell biological studies. Other applications are localized surface plasmon resonance (LSPR) or surface enhanced Raman spectroscopies (SERS), where specifically designed Au NPs areas on surfaces are indispensable because the surface plasmons of the Au NPs are affected by the distances among the particles by the overlapping of plasmon states of neighboring particles.

## Supplementary Materials

Supplementary Materials are available online at www.mdpi.com/2073-4360/9/6/199/s1.
