# Supplementary material for: Nano-Contact Transfer with Gold Nanoparticles on PEG Hydrogels and Using Wrinkled PDMS-Stamps
Source: Polymers (Basel). 2017 May 31;9(6):199. doi: 10.3390/polym9060199 (PMC6432311; doi:10.3390/polym9060199)
Supplement: Supplementary file 1 [file polymers-09-00199-s001.pdf]

Supporting Information

Nano-Contact Transfer with Gold Nanoparticles on PEG Hydrogels and Using Wrinkled PDMS-Stamps

Cigdem Yesildag, Arina Tyushina and Marga C. Lensen*

Technische Universität Berlin, Nanopatterned Biomaterials, Sekr. TC 1, Strasse des 17. Juni 124, 10623 Berlin, Germany; Lensen@Chem.TU-Berlin.de

* Correspondence: Lensen@Chem.TU-Berlin.de; Tel.: +49-30-314 29555

Academic Editor: Helmut Schlaad

Contents: In this supporting information, the following results are contained:

1) TEM images of Au NPs used and histogram of particle sizes
2) Control of wrinkle sizes by plasma treatment time
3) Transfer efficiency of the transfer of Au NPs from PDMS to PEG-hydrogel
4) Optical microscopy of (L929) fibroblast cells on Au NPs-patterned PEG-hydrogels

1) TEM images of Au NPs used and histogram of particle sizes

As-synthesized, citrate-stabilized gold nanoparticles (Au NPs) were characterized by Transmission Electron Microscopy (TEM). From software analysis of the TEM image (Figure S1; left), a size distribution histogram was derived (Figure S1; right) and the average particle size was determined to be 20.9 ± 2.5 nm.

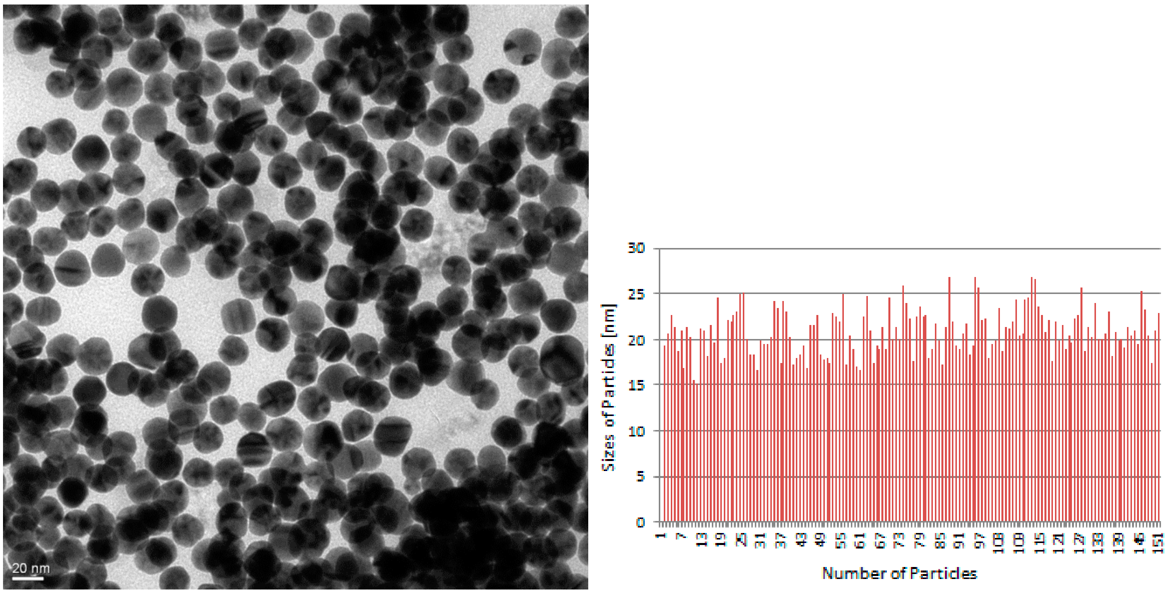

Figure S1: TEM image (left) and particle size distribution histogram (right) of the used Au NPs

2) Control of wrinkle sizes by plasma treatment time

From the repeatedly obtained AFM and SEM images for 2 min, 5 min and 15 min plasma treatment times, we prepared a statistical diagram (see Figure). As can be seen in the diagram,

a clear tendency is manifest: longer plasma treatment times result in larger distances between the wrinkles.

The relationship is however not linear; in the first few minutes the sizes increases rapidly and at longer treatment times the size increases more slowly, which is in correspondence with literature reports (N. Bowden, W. T. S. Huck, K. E. Paul, and G. M. Whitesides, "The controlled formation of ordered, sinusoidal structures by plasma oxidation of an elastomeric polymer," *Appl. Phys. Lett.*, vol. 75, no. 17, p. 2557, 1999.).

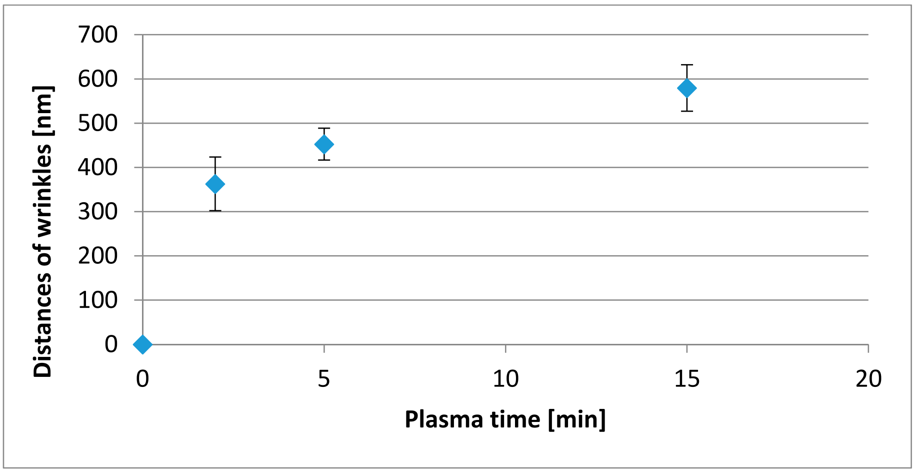

Figure S2: Distances of the wrinkles in relation with plasma treatment times

3) Transfer efficiency of the transfer of Au NPs from PDMS to PEG-hydrogel

The transfer efficiency was calculated with imageJ software via comparison of the number of Au NPs on PDMS before and after contact with PEG-hydrogel surface, according to the AFM images (Figure S3). A value of 50 ± 14 % for the still remaining Au NPs was obtained, thus approximately 50 % of the Au NPs are transferred to the surface of the PEG-hydrogel in one step.

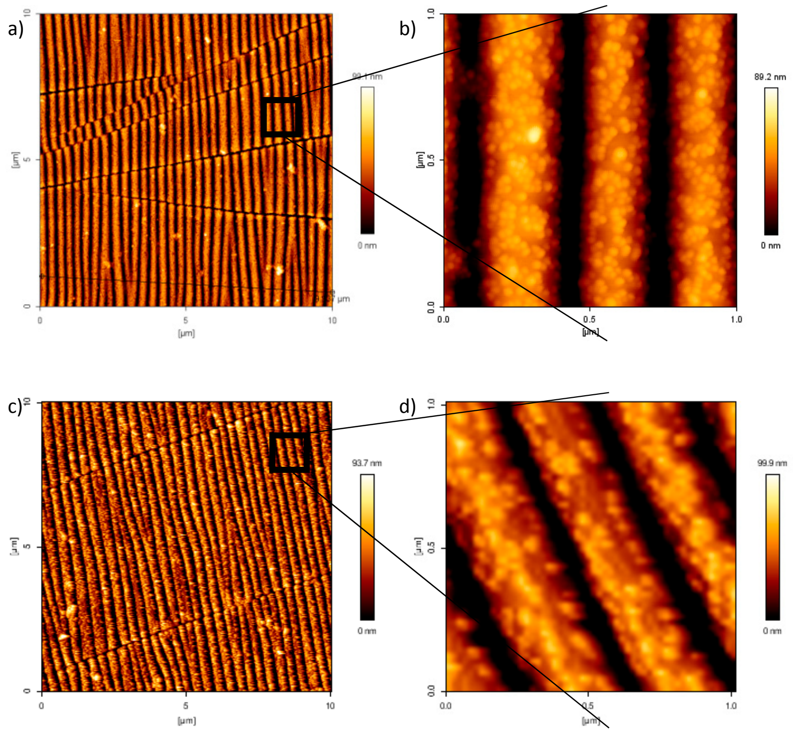

Figure S3: AFM images of the PDMS stamp; (a- b) before contact and (c-d) after contact with PEG-hydrogel.

4) Optical microscopy of (L929) fibroblast cells on Au NPs-patterned PEG-hydrogels

Due to the non-toxic, non-fouling (bio-inert) and non-immunogenic properties of the PEG-hydrogel and also the little toxicity of the Au NPs, these novel nanocomposite biomaterial can be applied in biological applications; such as for selective binding of proteins and enzymes and even whole cells. The optical micrographs of cells adhering to these nano-patterned hydrogels are shown in Figure S; the L929 cell adhesion on different surfaces is shown; a) on tissue culture polystyrene (TCPS) surface as control cells, b) on pure PEG-hydrogel and c) and d) on Au NPs nano-pattern on PEG-hydrogel:

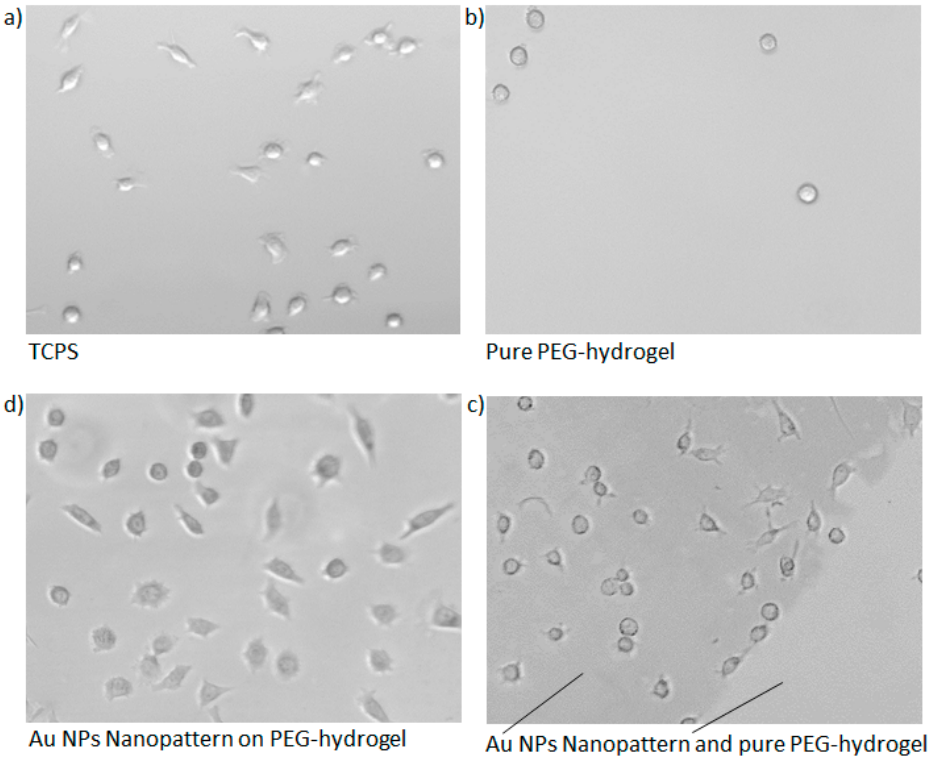

Figure S4: Optical micrographs of fibroblast L929 cell adhesion on: a) TCPS; b) pure PEG-hydrogel; c) Au NPs nano-pattern on PEG-hydrogel and c) a border of Au NPs nano-pattern and pure PEG-hydrogel.

It can be seen that the cells are not adhering on the pure PEG-hydrogel, they stay round shaped and only few cells are recognizable, which is due to the bio-inert property of the PEG-hydrogel. On the contrary, on the areas where Au NPs are present, cells do adhere and spread vividly; implying good viability.
